# Deep Neck Cellulitis, Myositis, and Reactive Lymphadenitis Secondary to Rothia dentocariosa in an Immunocompetent Female: A Case Report

**DOI:** 10.7759/cureus.66300

**Published:** 2024-08-06

**Authors:** Blake E Delgadillo, Mark A Potesta, Emma E Guld, Justin R Federico

**Affiliations:** 1 Orthopedic Surgery, Lake Erie College of Osteopathic Medicine, Bradenton, USA; 2 Internal Medicine, Lake Erie College of Osteopathic Medicine, Bradenton, USA; 3 Pediatrics, Lake Erie College of Osteopathic Medicine, Bradenton, USA; 4 Internal Medicine, Baptist Health, Jacksonville, USA

**Keywords:** virulent bacteria, infectious disease, gram-positive bacteria, bacteremia, immunocompetent host, reactive lymphadenitis, myositis, deep neck cellulitis, infective endocarditis, rothia dentocariosa

## Abstract

*Rothia dentocariosa* is a commensal organism that is typically found in the oropharyngeal and respiratory tracts, and it typically possesses a low virulence profile, especially for immunocompetent patients. The case presented here represents an extremely rare case of deep neck cellulitis, myositis, and reactive lymphadenitis secondary to *R. dentocariosa* in an immunocompetent female. A 35-year-old female with no significant past medical history presented to the emergency department with neck pain with reduced range of motion, fever, chills, sinus congestion, and headache for one day. After a thorough workup, blood cultures grew *R. dentocariosa* in the days following admission*.* The patient subsequently recovered without any notable sequelae after proper antibiotic treatment. Since *Rothia* species are currently considered a low-virulence organism that typically causes endocarditis in immunocompromised hosts, this case should serve as a reference for its possible virulence level in immunocompetent hosts. In spite of this organism's pathological rarity, this case highlights the importance of understanding the microbiology, historical context, and treatment for *R.* *dentocariosa* as a cause for deep neck cellulitis, myositis, and reactive lymphadenitis.

## Introduction

In 1967, the genus *Rothia* was first proposed and assigned to the family *Actinomycetacae* [1]. Similarities between *Actinomyces* and *Nocardia* were noted. However, independence was attained on the notion of chemotaxonomic, metabolic, and biochemical grounds [1].

*Rothia dentocariosa* is a gram-positive bacteria that is non-acid-fast, non-spore forming, non-pigmented, non-hemolytic, and non-motile [1]. Chemotaxonomically, it is characterized by a guanine-cytosine DNA ratio of 47-53% [1]. The cell wall sugars of *R. dentocariosa* are composed of ribose, glucose, fructose, and galactose [1]. The bacteria is also fermentative with lactic and acetic acid being the end-products [1]. Morphological variation, including coccoid, diphtheroid, and filamentous, has been noted in* R. dentocariosa* [1]. Additionally,* R. dentocariosa* may grow singly, in pairs, clusters, chains, or be pleomorphic [1].

*R. dentocariosa* is typically associated with the oropharynx and respiratory tract as a commensal organism [2]. Historically, this organism has been linked to periodontal disease [2]. Literature supports* R. dentocariosa* being considered low virulence, especially for immunocompetent patients [3]. Although this bacteria is typically not virulent in nature, the most common devastating sequelae have been documented to be endocarditis [3]. Still, only a few cases have been reported to date, with the first case causing endocarditis in 1978 [3]. In 2002, a literature review was performed of all 20 documented cases of infective endocarditis secondary to *R. dentocariosa*, and in 2011, another literature review was performed with only two new cases of infective endocarditis since the previous review [3]. Additionally, the Mayo Clinic in Rochester, USA, documented 25 cases of true positive blood cultures for* Rothia spp. *from 2002 to 2012 [4]. To the best of the authors’ knowledge, this is the first reported case of* Rothia spp.* deep neck cellulitis, myositis, and reactive lymphadenitis in an immunocompetent female.

## Case presentation

**Figure 1:**
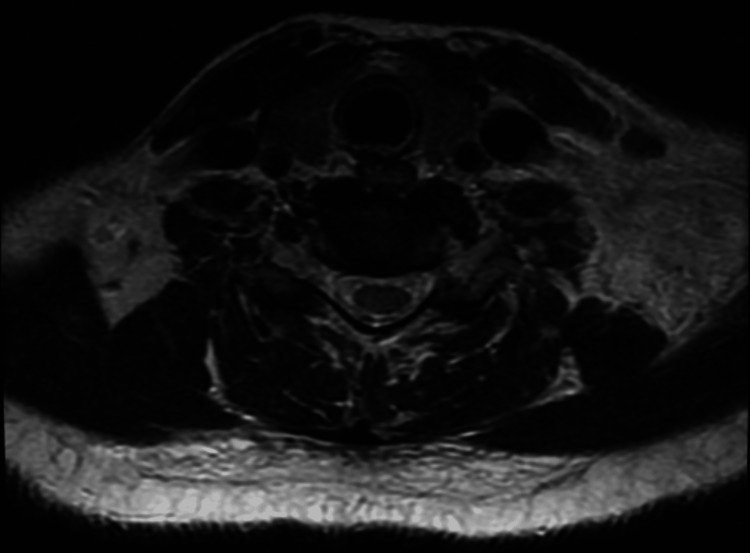
Transverse head and neck T2 magnetic resonance imaging Transverse head and neck T2 magnetic resonance imaging demonstrating left shoulder and supraclavicular edema extending into the left paravertebral soft tissues, left scalene, and left trapezius.

**Figure 2:**
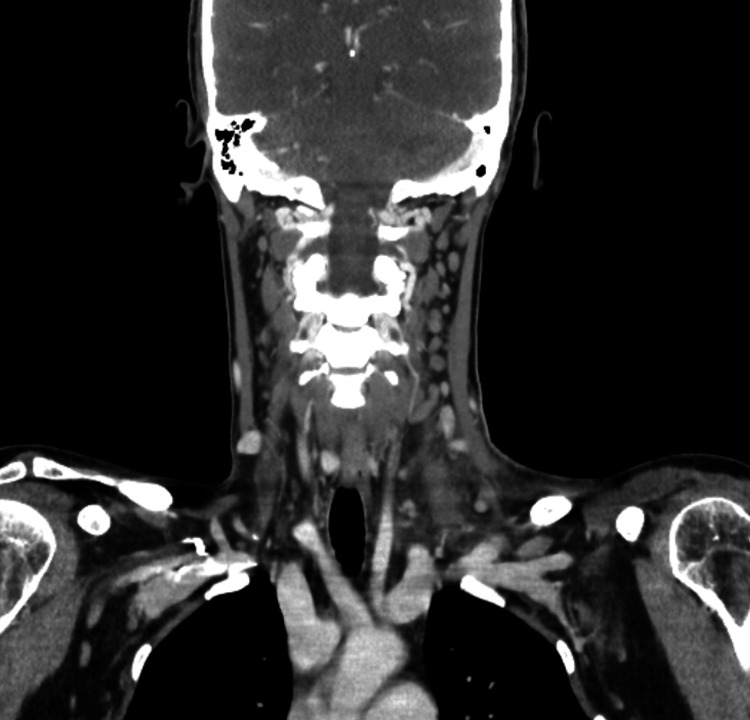
Coronal head and neck computed tomography with contrast Coronal head and neck computed tomography showing induration in the left lower neck and subclavicular fossa, extending into the left scalene triangle indicating cellulitis and myositis. Multiple enlarged reactive lymph nodes are appreciated. There is no evidence of abscess, fluid collection, or cervical spondylodiscitis.

A 35-year-old female with no significant past medical history presented to the emergency department with complaints of left anterior and lateral neck pain with reduced range of motion, fever, chills, diaphoresis, sinus congestion, and headache for one day. In the week prior to her presentation, she reported a brief bout of fishing in a nearby river and a mild, self-resolving upper respiratory infection. At that time, she had minor congestion and mild throat erythema without fever. Her immunizations were noted to be up-to-date. She denied cough, hemoptysis, sore throat, trismus, odynophagia, dysphagia, and ear pain. She also denied a history of deep vein thrombosis, oral contraceptive use, hormone therapy use, tobacco use, alcohol use, drug use, malignancy, and prior neck disease. The patient’s labs were unremarkable, with the exception of a leukocytosis of 18,000/mm^3^ (normal range: 4,500-11,000/mm^3^). The physical exam was notable for edema of the left anterior and lateral neck area with reduced range of motion of the cervical spine. There was pain with active and passive range of motion, and palpation of the area was tender to touch. There were no tonsillar exudates. Blood cultures were obtained, and she was placed on intravenous (IV) ceftriaxone and IV linezolid for suspected sepsis and cellulitis. Magnetic resonance imaging (MRI) demonstrated left shoulder/supraclavicular edema extending into the left paravertebral soft tissues, left scalene, and left trapezius without evidence of abscess formation (Figure 1). Computed tomography (CT) revealed evidence of cellulitis and myositis (Figure 2). Echocardiography revealed no valvular vegetation. After two days of growth, blood cultures grew gram-positive rods. Further analysis of blood cultures and repeat cultures identified *R. dentocariosa*, and IV doxycycline was added to her antibiotic regimen. The patient was subsequently diagnosed with deep neck cellulitis, myositis, and reactive lymphadenitis secondary to *R. dentocariosa*. Following treatment with IV antibiotics, the patient’s leukocytes were 10,200/mm^3^ after 48 hours, 6,800/mm^3^ after 72 hours, and 5,700/mm^3^ after 96 hours. Her discharge medications included doxycycline 100 mg per oral (PO), lactobacillus chewable tablet, and linezolid 600 mg PO. After finishing treatment, the patient subsequently recovered without any notable sequelae.

## Discussion

One of the challenges in diagnosing bacteremia secondary to *R. dentocariosa* is determining whether a positive blood culture is a true positive or a contaminant. According to a 10-year Mayo Clinic study on cases of *Rothia *bacteremia, 63% of cases studied were identified as potential contaminants if they grew from a single positive blood culture [4]. As a result, of the 67 positive cases of *Rothia*, 45 patients were excluded from the study, which left 25 patients to be deemed truly positive for *Rothia* bacteremia[4]. Of these 25 patients, 22 were neutropenic, and 3 were non-neutropenic [4]. Mayo Clinic’s decade-spanning study also concluded that gut translocation was the most commonly occurring source of infection in both neutropenic and non-neutropenic patients [4].

It is hypothesized that the reason gut translocation is the most common source of infection is due to antimicrobial disruption of the gut flora, which is most commonly secondary to chemotherapy-induced neutropenia and subsequent antimicrobial (levofloxacin) prophylaxis [4]. This is evidenced by 19 of the 22 neutropenic patients having a pre-existing diagnosis of leukemia and having received the aforementioned antimicrobial prophylaxis [4]. Additionally, in the three non-neutropenic patients, all patients had received antimicrobial treatment within the previous 30 days [4]. Following gut translocation, it was concluded that central line-associated bloodstream infections (CLABSI) and mucositis were the next most common sources of *Rothia* bacteremia [4]. Their study also indicated that monomicrobial or polymicrobial infections with* R. dentocariosa, Rothia mucilaginosa*, or other *Rothia* species led to no difference in clinical characteristics or outcomes [4]. Careful microscopic analysis and multiple positive blood cultures mitigated the risk of false positives [4].

Additionally, microscopic analysis presents a challenge as careful differential diagnosis from similar species must be considered. *Rothia* species shares some biochemical characteristics with *Actinomyces, Propionibacterium, *and* Corynebacterium*, such as being catalase-positive, aesculin-positive, nitrate-positive, and urease-negative [1]. To accurately identify *R.* *dentocariosa*, the following characteristics should be met: testing catalase-positive, aesculin-positive, nitrate-positive, and urease-negative, growing poorly under anaerobic conditions, testing negative for cyclic adenosine monophosphate, and testing positive for lactic acid as an end product (differing from succinate for *Actinomyces* and propionate for *Propionibacterium*) [1].

Other documented complications from *R. dentocariosa* have included cerebellar hemorrhages, cerebral embolism, intracranial hemorrhages, vertebral osteomyelitis, aortic root abscess, abdominal aneurysm, perivalvular abscess, and brain abscess [2]. Additionally, in the Mayo Clinic study, one non-neutropenic patient developed endocarditis, while another developed acute kidney injury [4]. Virtually all of the patients in the Mayo Clinic study had significant comorbidities [4]. Also, none of the patients in their study reportedly developed myositis, deep neck cellulitis, or reactive lymphadenitis, which is in contrast to the patient presented here. To the best of the authors’ knowledge, this is the first reported case of deep neck cellulitis, myositis, and reactive lymphadenitis secondary to *R. dentocariosa* in an immunocompetent patient with no significant past medical history.

## Conclusions

The patient in this case did not have antibiotic exposure within the previous 30 days of her positive blood cultures for *R. dentocariosa*, further highlighting the rarity of her presentation. This case is novel in several ways. First,* R. dentocariosa *bacteremia manifested in a non-neutropenic patient with atypical presenting symptoms including myositis, reactive lymphadenitis, and deep neck cellulitis. Additionally, there is uncertainty regarding the unidentified means of infection, as previously documented cases were almost universally associated with disruption of gut flora secondary to antimicrobials. The patient was successfully treated in an inpatient setting with a medication regimen consisting of IV ceftriaxone, IV linezolid, and IV doxycycline. This triad subsequently led to a marked improvement in her condition and the patient was discharged on a 10-day combination of doxycycline 100 mg PO, linezolid 600 mg PO, and a lactobacillus chewable tablet to promote healthy gut flora. The patient followed up with infectious disease two weeks later with complete resolution of her symptoms. This case should serve as a reference for *R. dentocariosa*’s potential virulence in non-neutropenic, immunocompetent hosts with no significant risk factors.
